# Development of short forms for screening children’s dental caries and urgent treatment needs using item response theory and machine learning methods

**DOI:** 10.1371/journal.pone.0299947

**Published:** 2024-03-22

**Authors:** Di Xiong, Marvin Marcus, Carl A. Maida, Yuetong Lyu, Ron D. Hays, Yan Wang, Jie Shen, Vladimir W. Spolsky, Steve Y. Lee, James J. Crall, Honghu Liu

**Affiliations:** 1 Section of Public and Population Health, Division of Oral and Systemic Health Sciences, School of Dentistry, University of California, Los Angeles, Los Angeles, California, United States of America; 2 Department of Biostatistics, Fielding School of Public Health, University of California, Los Angeles, Los Angeles, California, United States of America; 3 Division of General Internal Medicine and Health Services Research, Department of Medicine, David Geffen School of Medicine, University of California, Los Angeles, Los Angeles, California, United States of America; 4 Department of Health Policy and Management, Fielding School of Public Health, University of California, Los Angeles, Los Angeles, California, United States of America; 5 RAND Corporation, Santa Monica, California, United States of America; 6 Sectopm of Interdisciplinary Dentistry, Division of Diagnostic and Surgical Sciences, School of Dentistry, University of California, Los Angeles, Los Angeles, California, United States of America; University of Maribor, SLOVENIA

## Abstract

**Objectives:**

Surveys can assist in screening oral diseases in populations to enhance the early detection of disease and intervention strategies for children in need. This paper aims to develop short forms of child-report and proxy-report survey screening instruments for active dental caries and urgent treatment needs in school-age children.

**Methods:**

This cross-sectional study recruited 497 distinct dyads of children aged 8–17 and their parents between 2015 to 2019 from 14 dental clinics and private practices in Los Angeles County. We evaluated responses to 88 child-reported and 64 proxy-reported oral health questions to select and calibrate short forms using Item Response Theory. Seven classical Machine Learning algorithms were employed to predict children’s active caries and urgent treatment needs using the short forms together with family demographic variables. The candidate algorithms include CatBoost, Logistic Regression, K-Nearest Neighbors (KNN), Naïve Bayes, Neural Network, Random Forest, and Support Vector Machine. Predictive performance was assessed using repeated 5-fold nested cross-validations.

**Results:**

We developed and calibrated four ten-item short forms. Naïve Bayes outperformed other algorithms with the highest median of cross-validated area under the ROC curve. The means of best testing sensitivities and specificities using both child-reported and proxy-reported responses were 0.84 and 0.30 for active caries, and 0.81 and 0.31 for urgent treatment needs respectively. Models incorporating both response types showed a slightly higher predictive accuracy than those relying on either child-reported or proxy-reported responses.

**Conclusions:**

The combination of Item Response Theory and Machine Learning algorithms yielded potentially useful screening instruments for both active caries and urgent treatment needs of children. The survey screening approach is relatively cost-effective and convenient when dealing with oral health assessment in large populations. Future studies are needed to further leverage the customize and refine the instruments based on the estimated item characteristics for specific subgroups of the populations to enhance predictive accuracy.

## 1. Introduction

Early detection and intervention to prevent dental caries, the most common chronic disease of childhood [1], is critically important. In *Savage Inequalities*, Kozol noted that “Bleeding gums, impacted and rotting teeth are routine matters for the children I have interviewed in the South Bronx. The children get used to feeling constant pain” [2]. A recent study found that children with poor oral health status were three times more likely to miss school because of dental pain, with associated poorer school performance than their peers with better oral health [3]. Resource constraints substantially limit the feasibility of conducting traditional oral health examinations, especially for school-aged populations. The COVID-19 pandemic exacerbated the already limited access to school health education, nutritional support, and regular dental care [4, 5].

Rather than intervening in the destructive process of dental disease, it is more cost-effective to identify dental disease early and address urgent problems. Survey instruments have been developed to assess children’s oral health problems such as the Child Oral Impact on Daily Performances (Child-OIDP) [6], Early Childhood Oral Health Impact Scale (ECOHIS) [7], and the Child Perceptions Questionnaire (CPQ) [8, 9]. But these existing surveys focus on the oral health related quality-of-life impacts rather than on treatment referral recommendations. An instrument is needed that is accurate in identifying children who need treatment and at risk of long-term oral health problems.

Item response theory (IRT) is useful in identifying a parsimonious set of survey items with acceptable psychometric properties [10–13]. It has been widely used in education assessments [14], psychological tests [15], and health outcome measurements [16]. IRT models maximize the likelihood of the individual response pattern to estimate the item characteristics and the individual’s latent trait. Such item- and individual- information are crucial to understanding the survey performance and developing well-calibrated short forms. These item characteristic estimations also benefit further survey refinement, regardless of the number of item responses [17–19].

Machine Learning, on the other hand, is a data-centric approach to develop predictive and computationally efficient models. It has been used in dental research for disease identification [20, 21], image diagnosis [22], dental care and dental surgery needs [23]. A collection of classical Machine Learning algorithms have demonstrated value for classification and prediction. Similarly, as a regression model, other Machine Learning algorithms classify or predict outcomes based on predictors. These algorithms are designed under various principles or mechanisms, including tree-based methods (like CatBoost [24] and Random Forest [25]), probability-based methods (like Logistic Regression and Naïve Bayes), distance-based methods (like K-Nearest Neighbor [25] and Support Vector Machine [26]) and more complex Neural Networks [27]. Machine learning has also been used to select optimal subsets of survey items [28–31]. However, they can introduce selection bias and lead to overfitting issues due to selection primarily limiting targeted outcomes [11]. The best-performing subset identified by IRT is comparable to those selected using Machine Learning in terms of prediction power [11].

Prior work focused on the development of short forms associated with the Children’s Oral Health Status Index and referral recommendations based on Child Self-Reported Outcomes (CSROs) and Parent Proxy-Reported Outcomes (PPROs) using IRT [32, 33]. In addition, demographic information has been used in addition to short-form items to improve predictive performance using XGBoost and Naïve Bayes [28]. Dental caries among children has also been predicted using a Multivariate Adaptive Regression Spline [34]. None of this prior work used machine learning and IRT in combination to identify the best set of survey items.

Our work aims to fully utilize and synergize the strengths of IRT and Machine Learning to develop well-calibrated and efficient survey instruments for screening active caries and urgent treatment needs in school-age populations. Parsimonious instruments can be distributed by schools to facilitate routine oral health screenings for a quick evaluation of dental caries and treatment needs. Public health agencies and dental programs can use these short forms to monitor children’s oral health conditions and identify those at high risk regularly.

## 2. Methods

### 2.1. Source of data and participants

Our sample consisted of 497 dyads of children ages 8 to 17 and their parents. Families were recruited from 14 dental clinics and private practices across Los Angeles County in a cross-section study from August 2015 to October 2019. The participating sites provided dental care to children from low-to-high-income communities with a broad ethnic-racial diversity. The sample size was determined by using the standard error around a correlation which was approximately 0.045 [35] and was about sufficient for estimating the item response theory models [19].

The study excluded children who were in orthodontic treatment to avoid complexities and bias in performing tooth-based exam assessments. Only one child and parent per family was included. No specific treatments were administered during the study by the research team. However, children identified with additional dental care needs were referred to the clinics for evaluation and follow-up.

Institutional review board approval (#13–00130) was obtained from the Office of Human Research Protection Program, University of California, Los Angeles. Children and their parents signed written assent/consent forms before participating in the study.

### 2.2. Outcomes

All children received a dental examination to evaluate their clinical oral health status. Two experienced faculty dentists from UCLA Dental School performed the examinations following a Children’s Oral Health Protocol consisting of examinations on the overall occlusal condition and teeth status. Each primary and/or permanent tooth was recorded as being sound, decayed, missing, filled, bleeding, and with sealant.

The examiners conducted duplicated examinations on three students at each participating site. A total of 52 children were examined by both examiners on the same visit date to check the inter-rater reliability. The agreement was high using both Prevalence-Adjusted and Bias-Adjusted Kappa (0.77 for active caries and 0.8 for urgent treatment need) and Gwet’s AC1 (0.86 for active caries and 0.81 for urgent treatment need) [36, 37].

The clinical measures serve as the gold standard for calibration and evaluation of the oral health survey items. We focus on two dichotomous outcomes: whether the child 1) had at least one tooth with active caries (AC > 0) and 2) was in need for a dental reference and service (RFUTN). RFUTN was assigned using modified guidance from the National Health and Nutrition Examination Survey to focus on the severity of untreated decay, gingival bleeding (more than twelve teeth), and missing teeth due to caries [38].

### 2.3. Predictors

Before the clinical examinations of children, both children and their parents independently completed a self-administered computer-assisted survey about children’s oral health. The survey instrument was administered using the Questionnaire Development System, QDS^™^, (Nova Research Company, Bethesda, MD, USA) at the study clinics. Participants were required to answer all survey questions, resulting in complete data for this study with no missing responses.

Oral health survey items encompassed physical, mental, and social components based on a conceptual framework that reflected the complex nature of oral health [32, 33]. The survey assessed multiple aspects of children’s previous and current oral health status and behaviors such as overall oral health rating, teeth conditions, pain status, aesthetic, function limitations, experience recall (e.g., for the past 12 months, 4 weeks, 7 days, and 3 days), dental support, and so on (see S1 Data for the full list of items). Eighty-eight items for children and sixty-four items for parents were used for the analyses reported here. Demographic information was obtained using six child-reported and nine parent-reported items. The survey development process including focus groups, cognitive interviews, and full-item banks is discussed elsewhere [32, 35, 39]. In this paper, CSROs refer to Child Self-Reported Outcomes and PPROs for Parent Proxy-Reported Outcomes for better model labeling and comparison.

### 2.4. Statistical analysis methods

#### 2.4.1. Data preparation

CSRO and PPRO items associated with children’s oral health were rescaled into 0 to 5 with a higher score indicating worse oral health status, reflecting a higher likelihood of having a tooth with active caries and needing urgent dental care. If three or fewer responses were obtained for a response option, we collapsed this category with the adjacent higher-level option which represented a poorer oral health status. The full set of options was administered, but the collapsed options were used to estimate risk scores and make predictions. Highly right-skewed (positive skewness) items, as evidence of poor fit between the health status of the sample and the level of health measured by an item, were excluded [40]. Items that were positively and significantly associated (p-value < = 0.05 and r > = 0) or had at least a polychoric correlation > = 0.20 with the two clinical outcomes, the presence of active caries and urgent treatment needs, were further investigated to develop short forms.

#### 2.4.2. IRT assumptions

We first developed short forms using Samejima’s graded response model that estimates item thresholds and slope parameters for each ordered survey response item [41]. The item thresholds represent the trait level necessary to respond above threshold with a 50% chance of selecting a particular response option or a higher response option. A slope parameter represents the capability of this item to discriminate between contiguous latent trait levels. Before implementing the model, we evaluated the assumptions of “sufficient” unidimensionality, local independence, and monotonicity.

Unidimensionality indicates that the response to an item is accountable by one dominant latent trait. It can be assessed by single-factor confirmatory factor analysis (CFA) [42] using the checking comparative fit index (CFI > 0.95), Tucker-Lewis Index (TLI > 0.90), and the Root Mean Square Error of Approximation (RMSEA < 0.06) [43, 44]. CFA was conducted using an R package *Lavaan* [45] with polychoric correlations and the Mean- and Variance- Adjusted Weighted Least Square (WLSMV) robust estimations [45]. WLSMV estimations are more appropriate than maximum likelihood estimations for binary and ordinal variables [46, 47].

Local independence requires that the item responses are mutually independent when controlling for the underlying latent variable. For any pair of items with a residual correlation absolute value of 0.20 or higher in the single-factor CFA, the item with higher accumulated residual correlations was eliminated [40].

The monotonicity was evaluated by item characteristics curves to ensure the probability of endorsing a more severe response option should increase monotonically with the latent trait scores, such as the likelihood of active caries and RFUTN in this study.

#### 2.4.3. IRT calibration and differential item functioning analysis

IRT models were estimated using Mplus 8.3 [48] to obtain item thresholds and slopes and for maximum likelihood estimations of each child’s location on the underlying score continuum. The thresholds, or item difficulty, refer to the point on the latent trait scale at which there is a 50% chance of responding at or above a certain response level for each item; while the slop, or item discrimination, measures the ability of an item to differentiate between children with varying levels of oral health conditions.

Differential Item Functioning (DIF) was assessed for each item using ordinal logistic regression on estimated person scores for demographic subgroups (age group, gender, and parents’ education levels). All p-values are 2-sided with a significant level of 0.05. Multiple comparisons for DIF were assessed using Benjamini and Hochberg (BH) adjusted p-value to control for the false discovery rate [49, 50]. Ten-item short forms were selected with higher slope estimations, wider threshold parameters, and fewer DIF problems.

#### 2.4.4. Classification algorithms

The short-form and demographic items were combined to improve performance. All nominal variables were one-hot encoded with one dummy variable for each category. A collection of seven Statistical or Machine Learning algorithms was compared: CatBoost [24], Logistic Regression, K-Nearest Neighbors (KNN), Naïve Bayes, single-hidden-layer Neural Network [27], Random Forest [25], and Support Vector Machine (SVM) with Radial Kernels [26]. As there is no universal best algorithm for all data, each of these methods has its unique strengths in classification. The technical details of these classification algorithms are listed in the S1 Appendix.

To standardize the data for all algorithms, we performed the pre-process based on the training data first and the corresponding test set was projected onto the space of training data to test the developed models. We used the BoxCox transformation for continuous variables. All predictors were normalized to make the scale comparable using centering and scaling. The guideline of the transparent reporting of a multivariable prediction model for individual prognosis or diagnosis (TRIPOD) checklist [51] was followed as outlined in the S2 Appendix.

All algorithms, except for Logistic Regression, had a broad selection of hyperparameters that could influence the prediction performance (See S1 Appendix). Cross-validation (CV) helps to fine-tuned hyperparameters using a metric. The Receiver Operating Characteristic (ROC) curve plots the true positive rate (sensitivity) and the false positive rate (1 –specificity) given various classification thresholds. The Area under the ROC (AUC) could measure the model performance on each cross-validation testing set.

**Fig 1:**
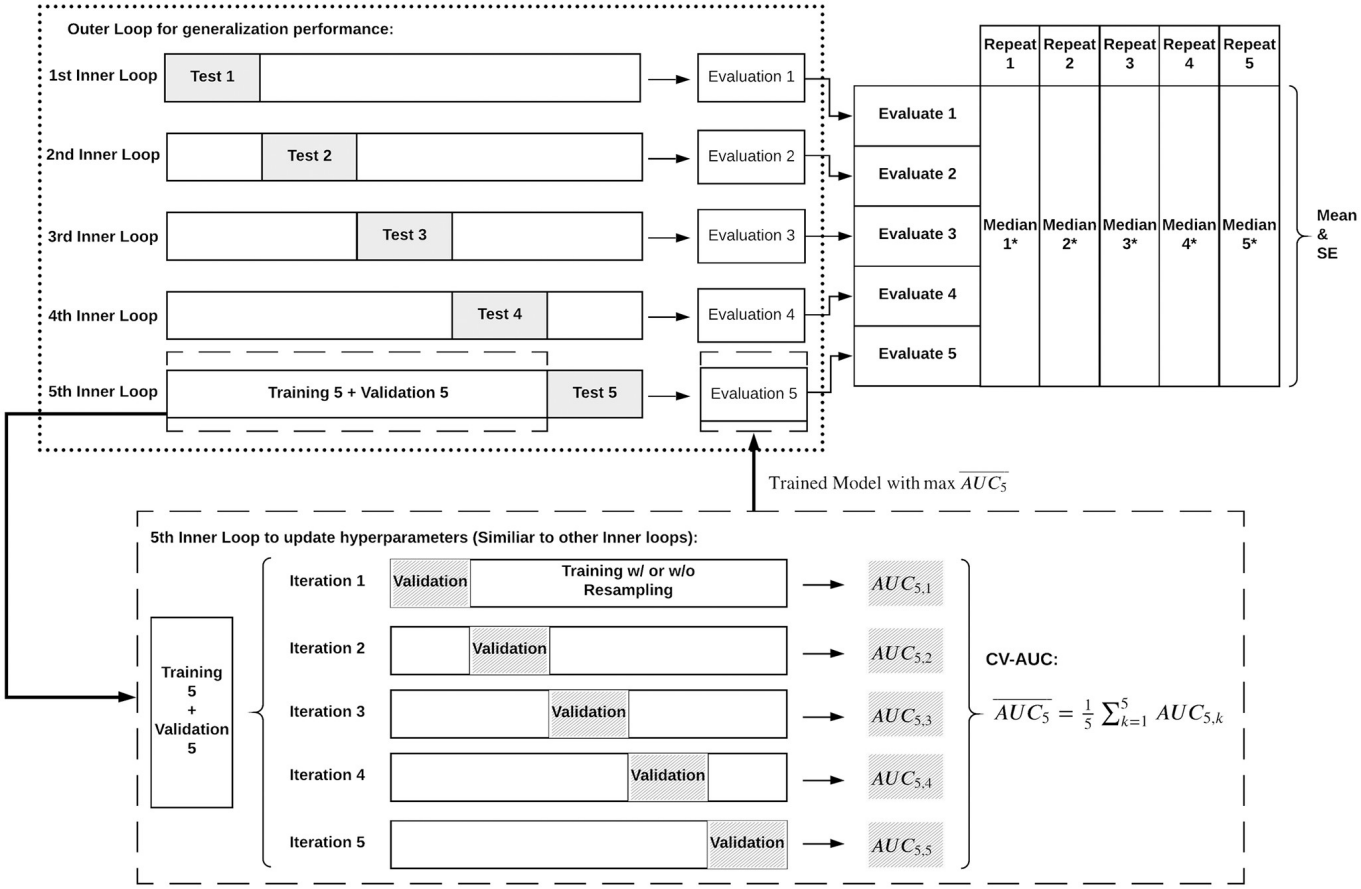
Repeated 5-fold nested cross-validation. (*: Median on performance metrics such as CV-AUCs, Sensitivity and Specificity on training-validation and testing sets).

To validate the performance efficiently on limited data and prevent overfitting, we repeated 5-fold Nested Cross-Validation (nCV) five times, as Fig 1. Each iteration within the inner loop utilized a fold for model validation and the rest for training. The models best-tuned by maximizing cross-validated AUC (CV-AUC) were evaluated on the corresponding testing set in the outer loop and CV-AUCs were aggregated.

Compared with the CV, nCV reduces bias of error estimation for the general performance, especially for sample sizes less than 1000 [52, 53]. The inner loop is responsible for tuning the hyperparameters, while the outer loop estimates the generalization accuracy. Means and standard errors of testing CV-AUC medians across five repetitions were used to select the best algorithm for each short form. Cutoff points for classification were chosen to maximize the sum of sensitivity and specificity and ensure sensitivity greater than 0.85 on training data. Summary statistics of accuracy performance metrics were calculated, including sensitivity, and specificity, precision, and f1 score, on both training-validation and testing sets.

Machine learning algorithms often face a common challenge of class imbalance, as most learning algorithms are initially designed for balanced data. The training subsets within inner-loop cross-validation could be resampled using Synthetic Minority Over-Sampling Technique (SMOTE) [54], which has been shown to perform better on imbalanced classification compared to other resampling methods [55]. In SMOTE, the majority class is randomly under-sampled by removing data; while the minority class is over-sampled by creating "synthetic" examples based on its K-nearest Neighbors instead of bootstrapping with replacement. The best algorithm for each short form was selected from 14 algorithms in the combinations of one of seven statistical or Machine Learning algorithms with and without SMOTE. The algorithms were implemented using *caret* and *DMwR* packages in R 3.6.3 [56–58].

#### 2.4.5. Model summary

The short-form development initiated with CSRO and PPRO items on children’s oral health and yielded four separate item pools to develop short forms related to 1) **AC-CSRO:** child self-reported active caries, 2) **RFUTN-CSRO:** child self-reported urgent treatment needs, 3) **AC-PPRO:** parents’ perception of their children having active caries, and 4) **RFUTN-PPRO:** parents’ perception of their children needing a referral for urgent treatment. We identified short-form items using IRT. Items that were verified by assumption checks were calibrated with estimations of slopes and thresholds, DIFs, and ability scores recorded.

The four short forms with demographic information were further enhanced using seven algorithms with and without SMOTE for better prediction accuracy. The refined short forms are 1) **AC-DEMO-CSRO**: AC-CSRO short-form items with children-reported demographic information; 2) **AC-DEMO-PPRO**: AC-PPRO short-form with parent-reported demographic information; 3) **AC-DEMO-CSRO-PPRO**: AC-CSRO and AC-PPRO short-form items with all available demographic information; and another three for RFUTN as 4) **RFUTN-DMO-CSRO**; 5) **RFUTN-DEMO-PPRO**; 6) **RFUTN-DEMO-CSRO-PPRO**. Using the repeated nested cross-validation method, we selected the best algorithm with the maximized mean of the testing CV-AUC medians for each of the refined short forms.

R and Mplus code for IRT assumption validation, short form calibration, machine learning model fitting, and generating tables and figures are available at https://github.com/dixiong777/COH_SF_IRTML.

## 3. Results

### 3.1. Participants

The sample included 497 dyads of children aged 8–17 years old and their parents with characteristics detailed in Table 1. This cross-sectional study design and on-site computer-assisted survey ensured a complete data set without any missing survey responses or clinical exam results. Approximately one-quarter (24.3%) of the children had at least one tooth with untreated decay. The RFUTN cases were the 42.3% of children who were identified as needed to see a dentist within the next 2 weeks (22.3%) or immediately (19.9%).

**Table 1:** Characteristics of children and parents (N = 497).

	Variables	Mean (SD) or No. (%)
**Clinical Outcomes**	Active Caries	
	No (AC = 0)	376 (76%)
	Yes (AC > 0)	121 (24%)
	Referral Recommendation	
	Continue your routine care	222 (45%)
	See a dentist at your earliest convenience	65 (13%)
	See a dentist within the next 2 weeks	111 (22%)
	See a dentist immediately	99 (20%)
**Child-reported Characteristics**	Children’s age, Mean (SD)	12 (2.9)
	Children age group^1^	
	8–12	290 (58%)
	13–17	207 (42%)
	Children’s gender	
	Male	255 (51%)
	Female	241 (49%)
	Female to male transgender	1 (0%)
	Children’s race and ethnicity	
	Caucasian/ White	87 (18%)
	Black/ African American	43 (9%)
	Hispanic/ Latino	227 (46%)
	Asian	54 (11%)
	Other	86 (17%)
	Children’s primary language	
	English	429 (86%)
	Other	68 (14%)
	Number of children in the household	
	1	177 (36%)
	2	165 (33%)
	3	79 (16%)
	> = 4	76 (15%)
**Parent-reported Characteristics**	Parent’s age, Mean (SD)	42.1 (8.8)
	Parent age group^1^	
	<30	40 (8%)
	30–44	262 (53%)
	45–59	179 (36%)
	> = 60	16 (3%)
	Parent’s gender	
	Male	131 (26%)
	Female	366 (74%)
	Children’s race and ethnicity	
	Caucasian/ White	78 (16%)
	Black/ African American	42 (9%)
	Hispanic/ Latino	242 (49%)
	Asian	46 (9%)
	Other	89 (18%)
	Parent’s race and ethnicity	
	Caucasian/ White	103 (21%)
	Black/ African American	42 (9%)
	Hispanic/ Latino	258 (52%)
	Asian	56 (11%)
	Other	38 (8%)
	Parent’s primary language	
	English	318 (64%)
	Other	179 (36%)
	Child has dental insurance	
	No	104 (21%)
	Yes	393 (79%)
	Parental employment status	
	Full-time job	382 (77%)
	Part-time job	54 (11%)
	Not working	61 (12%)
	Parent’s Marriage Status	
	Married/ Living with Partner	370 (74%)
	Single	127 (26%)

^1^Characteristics not used for the prediction models.

### 3.2. Model Development and specification

#### 3.2.1. Data Preparation and IRT assumptions

After we checked the correlation with the target outcomes, we selected the screening item pools for **AC-CSRO** (17 items), **RFUTN-CSRO** (27 items), **AC-PPRO** (27 items), and **RFUTN-PPRO** (19 items). Two highly skewed items (skewness = 9.79, “tobacco use” for **RFUTN-CSRO,** and “hospital emergency needs in the last 12 months” for both **AC-PPRO** and **RFUTN-PPRO**) were further excluded.

Single-factor CFAs were estimated for the remaining 17 items for **AC-CSRO**, 26 items for **RFUTN-CSRO**, 26 items for **AC-CSRO,** and 18 items for **RFUTN-PPRO** at first. The initial fit indices for all four models were: **AC-CSRO** (CFI = 0.927, TLI = 0.916, RMSEA = 0.061), **RFUTN-CSRO** (CFI = 0.924, TLI = 0.918, RMSEA = 0.051), **AC-PPRO** (CFI = 0.852, TLI = 0.839, RMSEA = 0.070), and **RFUTN-PPRO** (CFI = 0.894, TLI = 0.880, RMSEA = 0.065).

For each pair of correlated items, items with higher accumulative correlations were considered as being locally dependent including three items for **AC-CSRO**, seven items for **RFUTN-CSRO**, five items for **AC-PPRO,** and nine items for **RTFN-PPRO**. For example, for **RFUTN-CSRO**, “peer jokes on how teeth look” was eliminated due to high residual correlations with five items ("miss school days”, “being afraid to see a dentist”, “brush teeth” “cognition of flossing teeth”, “parents as important people to oral health”, “medical providers as important people to oral health”).

With these local-dependent items removed, the final fit indices improved and produced adequate fit for all four models: **AC-CSRO** (12 items, CFI = 0.992, TLI = 0.990, RMSEA = 0.029), **RFUTN-CSRO** (19 items, CFI = 0.970, TLI = 0.966, RMSEA = 0.045), **AC-PPRO** (17 items, CFI = 0.952, TLI = 0.944, RMSEA = 0.057), and **RFUTN-PPRO** (15 items, CFI = 0.981, TLI = 0.978, RMSEA = 0.039). More details on the assumption checking are in S1 Data.

#### 3.2.2. IRT calibration and DIF analysis

The remaining items were calibrated to estimate item threshold and slope parameters (See more details in S2 Data). Ten-item short forms were selected with higher slope estimations, wider threshold parameters, and fewer flags for DIFs from the corresponding long forms, as presented in Table 2.

**Table 2:** Item calibration statistics.

**a. 10-item AC-CSRO Short Form**						
**Items**	**Slope** ^a^	**Location Thresholds**				
		1	2	3	4	5
It was hard for me to have fun because of the pain in my mouth.	4.34	5.92	7.79	9.95		
It was hard for me to pay attention because of the pain in my mouth.	4.17	5.01	7.03	9.93		
I felt stressed because of the pain in my mouth.	3.86	4.94	6.60	9.05		
It was hard for me to talk because of the pain in my mouth.	2.86	4.05	5.94	7.36		
It was hard for me to eat because of the pain in my mouth.	2.51	2.21	4.04	5.65	6.47	
In the last 4 weeks, how much of the time did you have pain or discomfort?	1.50	0.86	2.17	3.81	4.66	
In the last 4 weeks, how much of the time were you able to swallow comfortably?	0.75	1.21	1.71	1.91	2.29	2.53
Did any of the following reasons ever keep you from visiting a dentist? My parents didn’t have any money for a dentist.	0.49	2.71				
How much are you afraid to go to a dentist?	0.41	0.33	2.05	3.06		
How often do you brush your teeth?	0.38	0.50	2.26	2.95	3.28	
**b. 10-item RFUTN-CSRO Short Form**						
**Items**	**Slope** ^a^	**Location Thresholds**				
		1	2	3	4	5
I felt stressed because of the pain in my mouth.	4.58	5.74	7.52	10.02		
It was hard for me to pay attention because of the pain in my mouth.	4.29	5.84	7.56	9.49		
It was hard for me to have fun because of the pain in my mouth.	4.31	5.17	7.10	9.79		
It was hard for me to sleep because of the pain in my mouth.	3.38	4.69	6.32	8.28		
It was hard for me to eat because of the pain in my mouth.	2.85	2.45	4.38	6.03	6.85	
It was hard for me to talk because of the pain in my mouth.	3.03	4.22	6.08	7.44		
It hurts my teeth to chew.	2.06	2.14	3.82	6.06		
I had a tooth that hurts.	1.91	1.44	2.75	4.62	5.86	
My mouth hurts.	1.95	1.32	3.30	5.74		
During the last school year, how many days of school did you miss because of pain in the mouth, tongue, teeth, jaws, or gums?	1.20	3.31	4.24	1.20		
**c. 10-item AC-PPRO Short Form**						
**Items**	**Slope** ^a^	**Location Thresholds**				
		1	2	3	4	5
It was hard for my child to eat because of the pain in his/her mouth.	4.36	4.73	7.30	9.76		
It hurts my child’s teeth to chew.	3.69	3.92	6.26	8.60		
It was hard for my child to talk because of the pain in his/her mouth.	3.63	5.70	7.67			
My child’s mouth hurts.	2.93	2.60	5.16	7.69		
My child’s gums hurt.	2.92	3.47	5.26			
My child had a tooth that hurts.	2.78	2.41	4.10	7.037		
My child’s jaw hurts.	2.59	3.88	5.57			
During the last school year, how many days of school did your child miss because of pain in his/her mouth, tongue, teeth, or gums?	1.30	3.57	4.33			
During the last 12 months, did your child have an oral health problem?	0.93	1.23				
In the last 4 weeks, how much of the time were you pleased or happy with the look of your child’s mouth, teeth, jaws, or gums?	0.54	-0.66	0.86	1.53	2.82	3.38
**d. 10-item RFUTN-PPRO Short Form**						
**Items**	**Slope** ^a^	**Location Thresholds**				
		1	2	3	4	5
It was hard for my child to eat because of the pain in his/her mouth.	3.90	4.14	6.64	9.13		
My child had a tooth that hurts.	3.24	2.63	4.54	7.95		
My child’s gums hurt.	2.59	2.32	4.71	7.21		
My child’s mouth hurts.	2.60	3.10	4.82			
During the last school year, how many days of school did your child miss because of pain in his/her mouth, tongue, teeth, or gums?	1.29	3.56	4.32			
During the past 12 months, was there a time that your child needed dental care but did not get it?	1.01	2.85				
In the last 4 weeks, how much of the time were you pleased or happy with the look of your child’s mouth, teeth, jaws, or gums?	0.70	-0.68	0.90	1.59	2.91	3.47
How much is your child afraid to go to a dentist?	0.56	0.40	1.71	3.15		
Compared to other kids my child’s age:	0.43	-0.07	3.19			
How often do you remind your child to brush his/her teeth before he/she goes to sleep?	0.28	-3.01	-1.93	-0.88	-0.47	0.12

^a^Items are ordered based on the size of their slopes.

Short form items encompass various aspects, covering physical, mental, and social domains. Items concerning enjoyment, attention, and stress difficulties due to the pain had higher slopes and thresholds in CSRO short forms for both outcomes. It indicated that they were sensitive to distinguishing among children with poorer oral health. The AC-CSRO short form quired more questions related to functioning challenges due to oral health issues, while RFUTN-CSRO short form focuses on recent direct pain. Unlike these children short forms, in the parent short forms, the item about eating difficulty was more effective to identify children at risk with the highest slope and high location thresholds. These parent short forms included with more long-term pain questions. Comparing with the AC-PRRO short form, RFUTN-PPRO contains items on oral healthcare access, children’s fear of dental visit, and brushing habits, which were also in the AC-CSRO short form for children response.

The information curves for the short forms and their corresponding long forms are shown in S1 Fig.

#### 3.2.3. Classification

The calibrated short-form items were combined with demographic items listed in Table 1. Our approach employing seven algorithms, each with and without SMOTE, through 5-fold nCV. The validations were repeated 5 times independently, resulting in 350 local fine-tuned best models using traditional 5-fold Cross-Validation (as 5 best-tuned models by 5-fold CV-AUC × 7 algorithms × 2 resampling options × 5 times) for each refined short-form. The prediction models were evaluated and compared, as detailed in Table 3, to assess the classification performances of the short forms.

**Table 3:** Estimated mean and standard errors of nCV-AUC median.

Refined Short Form^a^	Resampling	CatBoost	Logistic Regression	K-Nearest Neighbors	Naïve Bayes	Neural Network	Random Forest	SVM with Radial Kernel
AC-DEMO-CSRO	No	0.61 (0.02)	0.59 (0.01)	0.61 (0.01)	**0.62 (0.00)**	0.60 (0.01)	0.61 (0.01)	0.60 (0.00)
	SMOTE	0.59 (0.01)	0.57 (0.01)	0.61 (0.01)	0.60 (0.01)	0.60 (0.01)	0.57 (0.01)	0.58 (0.01)
AC-DEMO-PPRO	No	0.60 (0.01)	0.57 (0.02)	0.58 (0.01)	**0.63 (0.00)**	0.58 (0.01)	0.60 (0.02)	0.59 (0.01)
	SMOTE	0.58 (0.01)	0.55 (0.01)	0.60 (0.01)	0.60 (0.01)	0.58 (0.01)	0.58 (0.01)	0.56 (0.01)
AC-DEMO-CSRO-PPRO	No	0.60 (0.01)	0.56 (0.00)	0.58 (0.01)	**0.64 (0.01)**	0.58 (0.01)	0.62 (0.01)	0.60 (0.01)
	SMOTE	0.58 (0.00)	0.55 (0.01)	0.59 (0.01)	0.56 (0.02)	0.57 (0.02)	0.58 (0.02)	0.56 (0.02)
RFUTN-DEMO-CSRO	No	0.58 (0.00)	0.56 (0.01)	0.59 (0.01)	0.58 (0.01)	0.57 (0.01)	0.56 (0.01)	0.60 (0.01)
	SMOTE	0.57 (0.01)	0.54 (0.01)	0.57 (0.00)	**0.60 (0.01)**	0.57 (0.01)	0.54 (0.01)	0.59 (0.01)
RFUTN-DEMO-PPRO	No	0.60 (0.01)	0.61 (0.00)	0.59 (0.01)	**0.62 (0.00)**	0.60 (0.01)	0.59 (0.01)	0.61 (0.01)
	SMOTE	0.58 (0.01)	0.59 (0.01)	0.57 (0.01)	0.61 (0.01)	0.60 (0.01)	0.57 (0.01)	0.57 (0.00)
RFUTN-DEMO-CSRO-PPRO	No	0.60 (0.01)	0.58 (0.01)	0.60 (0.01)	**0.62 (0.01)**	0.60 (0.01)	0.60 (0.01)	0.61 (0.01)
	SMOTE	0.59 (0.01)	0.57 (0.01)	0.57 (0.01)	0.61 (0.02)	0.59 (0.01)	0.58 (0.01)	0.57 (0.01)

^a^ The best algorithm for each refined short form is in bold.

### 3.3. Model performance

Naïve Bayes algorithm without SMOTE outperformed other algorithms for all refined short forms with higher nCV-AUC medians, except one (**RFUTN-DEMO-CSRO**) of which the best model was still Naïve Bayes but with SMOTE. Their prediction performance, including sensitivity, specificity, precision, and F1 score, on training-validation and testing sets for each refined short form are presented in Table 4. Models incorporating both self-reported and proxy-reported responses showed a slightly higher predictive accuracy than those relying on either child-reported or proxy-reported responses.

**Table 4:** Prediction performance on each refined short form based on the best algorithm.

Refined Short Form	Training-validation Set				Testing Set			
	Sensitivity	Specificity	Precision	F1 Score	Sensitivity	Specificity	Precision	F1 Score
AC-DEMO-CSRO	0.87 (0.01)	0.33 (0.05)	0.30 (0.01)	0.45 (0.01)	0.83 (0.02)	0.29 (0.02)	0.27 (0.00)	0.42 (0.01)
AC-DEMO-PPRO	0.86 (0.01)	0.36 (0.02)	0.30 (0.01)	0.45 (0.01)	0.85 (0.06)	0.31 (0.03)	0.27 (0.01)	0.42 (0.01)
AC-DEMO-CSRO-PPRO	0.87 (0.01)	0.35 (0.03)	0.31 (0.00)	0.45 (0.00)	0.84 (0.05)	0.30 (0.01)	0.27 (0.00)	0.42 (0.00)
RFUTN-DEMO-CSRO	0.86 (0.01)	0.42 (0.04)	0.46 (0.01)	0.61 (0.01)	0.77 (0.04)	0.36 (0.04)	0.44 (0.01)	0.57 (0.02)
RFUTN-DEMO-PPRO	0.87 (0.02)	0.24 (0.02)	0.51 (0.01)	0.64 (0.01)	0.81 (0.03)	0.18 (0.03)	0.47 (0.00)	0.59 (0.01)
RFUTN-DEMO-CSRO-PPRO	0.87 (0.03)	0.39 (0.02)	0.52 (0.01)	0.65 (0.01)	0.81 (0.06)	0.31 (0.05)	0.47 (0.01)	0.59 (0.01)

Among 350 local fine-tuned CVs for each refined short form, models with the best testing performance were generated by different algorithms: KNN with SMOTE for **AC-DEMO-PPRO** (sensitivity = 0.84, specificity = 0.48), and **AC-DEMO-CSRO-PPRO** (sensitivity = 0.83, specificity = 0.45), **RFUTN-DEMO-CSRO-PPRO** (sensitivity = 0.83, specificity = 0.45); Radial Kernel SVM without SMOTE for **AC-DEMO-CSRO** (sensitivity = 0.83, specificity = 0.53), and **RFUTN-DEMO-CSRO** (sensitivity = 0.81, specificity = 0.53); CatBoost without SMOTE for **RFUTN-DEMO-PPRO** (sensitivity = 0.83, specificity = 0.52).

## 4. Discussion

This study aims to integrate the capabilities of IRT and machine learning to build survey short forms and predictive instruments for active caries and urgent treatment needs screening in large school-age populations.

We developed ten-item short forms for active caries and referral for urgent treatment needs based on children self-reported and parent proxy-reported information separately using IRT. The slopes and thresholds were the key parameters used for selecting variables that were independent of outcomes. These short forms provide high discriminability and wide thresholds to cover children with various health conditions.

Short forms based on child reports only, parent reports only, and child and parent reports collectively classified children using multiple statistical and machine learning algorithms. The Naïve Bayes outperformed other algorithms in general. For refined short forms using all CSRO and PPRO items, the average testing sensitivity and specificity were 0.84 and 0.30 for active caries, and 0.81 and 0.31 for RFUTN. The corresponding best testing sensitivities and specificities for local fine-tuned models were both 0.83 and 0.45 using KNN with SMOTE.

### 4.1. Study limitations

Although we included a diverse sample, it was a convenient sample drawn from children with a dental home, which does not represent the general school-age population. Moreover, the prediction performance of the short forms appears to be weak given the current samples. The classification accuracies increase with the increment of samples when the datasets have a good discriminative power between two classes [59]. Our survey questions about children’s oral health perceptions covered aspects of dental caries, missing teeth, and periodontal diseases, but they were not disease-specific. Parents and children responded to the surveys based on their perceptions of oral health, especially in the past three months or the last 12 months, which might affect concordance with oral exam results. In addition, some oral diseases are likely asymptomatic at an early disease stage and cannot be identified through self-reported survey questions.

To improve the survey protocol of oral health screening for school-age children, our ongoing work involves a more representative sample, more disease-specific survey questions, and the inclusion of various additional data sources.

### 4.2. Interpretation

Self-reported enjoyment, attention and stress challenges due to pain were more effective in detecting children with active caries and urgent dental needs. It was more obvious to children to have a dental cavity when the pain caused functioning issues or need urgent dental care when they experienced recent pain; Parents, in contrast, identified kids’ oral health issues based on more long-term evidences. The predictive models showed a slightly higher predictive accuracy when combining child-reported or proxy-reported responses.

The short-form items in the short forms for the current study overlapped with most of the items identified in previous work on active caries, 60% for the children survey and 67% for the parent survey [34]. In addition to different sample sizes, the two studies used different optimization principles to select items. The previous study optimized prediction accuracy only, while this study also considered the interaction of survey items and responder’s underlying latent trait simultaneously using IRT, which is better for making predictions on general populations [11].

To investigate the general performance, we repeated algorithms five times for this study. The Repeated 5-fold Nested Cross-Validation helped to reduce the random uncertainty due to model instability and improved model accuracy while not solving overfitting issues completely. Additional external validation on new samples is also needed to ensure the reliability and validity of the developed short forms for the large population.

Because it is critical to identify patients with true active caries or treatment needs correctly for large populations, we traded off the cutoff point of classification thresholds to maintain a sensitivity at least of 85% in the training data sets, resulting in lower specificity rates. This paper only evaluated whether SMOTE resampling technique can help to improve the performance for this use case. In addition to the improve sample data quality, we can also implement other techniques, such as weighted class, under-sampling, data augmentations, and other resampling technique variants, to improve the classification performance for imbalanced class data.

The predictive accuracy can be further enhanced when integrated with other types of data, even with small sample data. A caries risk prediction model using a random forest achieved a test AUC as 0.73 based on oral health questionnaire responses and genetic markers, Single nucleotide polymorphisms (SNPs) [60]. With previous caries history and acidogenicity of dental biofilms, the test AUC of the caries risk models was 0.78 [61].

### 4.3. Implications

The oral health assessment short forms have highly significant value in potentially overcoming longstanding public health challenges in oral health assessment for large populations of children. Such screening supplement of the clinical examinations, yet to be highly accurate, can help to identify the sub-cohort children who are most vulnerable. Schools and families can more effectively monitor oral health changes. Additional notable strengths include (i) ability to implement through electronic platforms, such as a computer or mobile device, and easy use in large populations; (ii) reduces the need for oral health professionals, thereby lowering cost and making surveillance more feasible and sustainable; (iii) ability to be used by a wide range of individuals and in a wide range of settings; (iv) reduced risk of disease transmission from person-to-person contact, thereby allowing for surveillance during COVID-19 or other future pandemics.

In our continued efforts to improve the classification accuracy, especially on low specificities, we will refine the survey based on the current findings on item characteristics and prediction performance. We will target to more specific dental diseases of interest with direct oral health questions, such as teeth observations, home relief medications, and treatment history. Furthermore, other types of study design and data collection methods have also been considered for our future work. Due to the dynamic dental disease status, it will be more useful to provide a more precise recall interval. Compared to the cross-sectional study, the longitudinal study is a better option to validate the recall referral recommendation and track the progression of oral health diseases [62, 63]. Moreover, linking the survey protocol to other data sources, especially intraoral images [16, 54, 55], is expected to boost screening accuracy with machine learning and deep learning algorithms.

## 5. Conclusions

This study developed short forms to identify children who have active caries and/or urgent treatment needs. The proposed framework for short-form development integrates the strength of both IRT and machine learning to enhance clinical decision support. The short forms and this line of research hold the potential to overcome challenges associated with oral health screening for large populations of school-age children.

## Supporting information

S1 Data Aggregated data for assumption checking. (XLSX)

S2 Data Item calibration statistics for long forms. (XLSX)

S1 Fig Information curves of long-form and short-form for AC and RFUTN models. (DOCX)

S1 Appendix Technical details of the classification algorithms. (DOCX)

S2 Appendix TRIPOD checklist. (DOCX)

S3 Appendix List of abbreviations. (DOCX)
