# Supplementary material for: Development of short forms for screening children’s dental caries and urgent treatment needs using item response theory and machine learning methods
Source: PLoS One. 2024 Mar 22;19(3):e0299947. doi: 10.1371/journal.pone.0299947 (PMC10959356; doi:10.1371/journal.pone.0299947)
Supplement: S1 Fig — (DOCX) [file pone.0299947.s003.docx]

# **S1 Figure. Information Curves of Long-form and Short-form for AC and RFUTN Models**

**
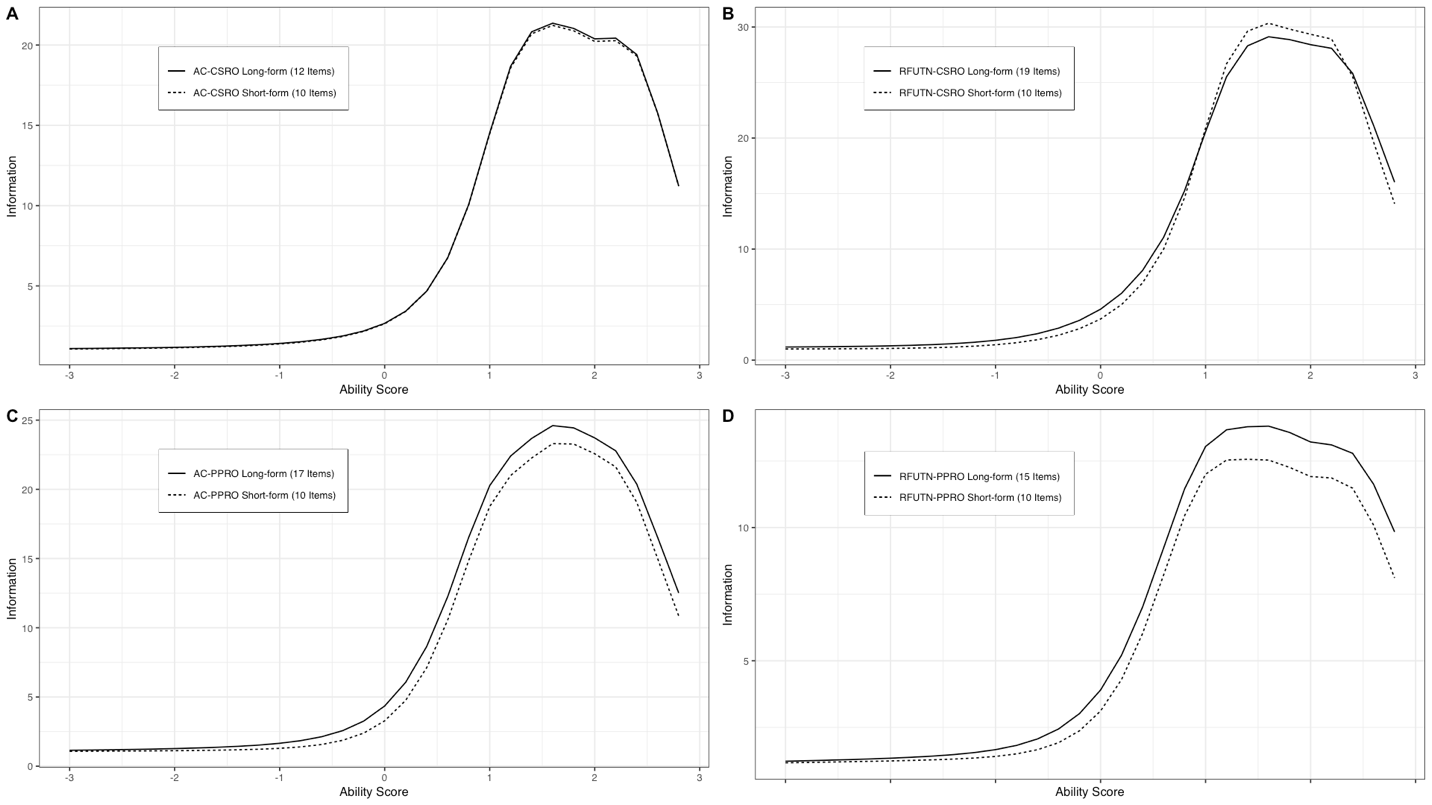
**

Short forms preserved the shape of that of the long forms with area under the information curve reduced by 0.7% for AC-CSRO, 1.9% for RFUTN-CSRO, 8.9% for AC-PPRO, and 10.9% for RFUTN-PPRO, which indicates that selected short forms were consistent with long forms. The RFUTN-CSRO short form has a higher point-estimated information values than that of long form.
