# Supplementary material for: Development of short forms for screening children’s dental caries and urgent treatment needs using item response theory and machine learning methods
Source: PLoS One. 2024 Mar 22;19(3):e0299947. doi: 10.1371/journal.pone.0299947 (PMC10959356; doi:10.1371/journal.pone.0299947)
Supplement: S1 Appendix — (DOCX) [file pone.0299947.s004.docx]

# **S1 Appendix. Technical Details of the Classification Algorithms**

A total of seven algorithms have been investigated, including CatBoost, Logistic Regression, K-Nearest Neighborhood (KNN), Naïve Bayes, single-hidden-layer Neural Network, Random Forest, and Support Vector Machine (SVM) with Radial Kernels.

The *caret* package (version 6.0-86 used in the manuscript) in R provides a comprehensive framework to build and validate various machine learning models with support on data splitting, pre-processing, resampling, model fitting, cross-validation, and testing.

The training-testing and cross-validation data splitting in each replication of nested cross-validation were consistent across all algorithms. To standardize the data for all algorithms, we performed the pre-process based on training data at first and the corresponding test set was projected onto the space of training data while testing the developed models. The BoxCox transformation on the continuous variables helped to transform the data into a better normal shape. All predictors were normalized to make the scale comparable using centering and scaling. Then, we identified key independent components that explained 95% of the variance using principal component analysis in the pre-processing step. We also incorporated Synthetic Minority Over-Sampling Technique (SMOTE) within cross-validation for the imbalanced data using *DMwR* package (version 0.4.1).

In addition, a collection of packages is required to implement some algorithms, such as *catboost* (version 0.24.4), *klaR* (version 0.6-15), *nnet* (version 7.3-15), and *randomForest* (version 4.6-14). The algorithms might use different estimation approaches for categorical and continuous dependent variables. Naïve Bayes, for example, needs to estimate the conditional probability P(C = c | X = x) where C is the class outcome, and X is a dependent variable. For the categorical data, it directly computed the conditional probability of the outcomes given each categorical level of each dependent variable; while for the continuous data, it made a normal approximation to estimate the P(X = x |C = c) first and then used Bayes Rule to estimate the conditional probability needed for the classifier.

All algorithms, except for logistic regression, had a broad selection of hyperparameters that can influence the prediction performance. The tunning hyperparameters are listed in the following table. All other parameters were left at their default values. For example, the Catboost algorithm grew a total of 1000 symmetric trees at most with the depth of 6, at least 1 subject in each leaf and at most 31 leaves per tree; the learning rate for reducing gradient step is 0.03; Coefficient at the L2 regularization term of the cost function was set as 3.0 and early stopping was allowed.

# **Table. List of Tuning Hyperparameters Used**

| **Machine Learning Algorithm** | **Hyperparameters** | **Tuning Range** | **Number of Variants** |
| --- | --- | --- | --- |
| **CatBoost** | depth: depth of the tree. | 2, 4, 6 | 12 |
|  | learning_rate: learning rate to reduce the gradient steps. | 0.05, 0.14, 0.37, 1.0 |  |
| **K-Nearest Neighborhood** | k: the number of neighbors "voting" | 5, 6, 7, 9, 11, 13, 15, 17, 21, 23 | 10 |
| **Naïve Bayes** | useKernel: whether to use a kernel density estimate | True, False | 12 |
|  | adjust: the bandwidth of the kernel density (larger numbers mean a more flexible density estimate) | 0, 1, 2, 3, 4, 5 |  |
| **Neural Network** | size: number of units in the hidden layer | 1, 3, 5 | 9 |
|  | decay: ﻿parameter for weight decay | 0, 0.0001, 0.1 |  |
| **Random Forest** | mtry: Number of variables available for splitting at each tree node | 2, 6, 10 | 3 |
| **Support Vector Machine with Radial Kernel** | C: cost for the possible misclassification | $2^{i-2}, i=0, 1, \ldots, 9$ | 10 |
