# Supplementary material for: Development of short forms for screening children’s dental caries and urgent treatment needs using item response theory and machine learning methods
Source: PLoS One. 2024 Mar 22;19(3):e0299947. doi: 10.1371/journal.pone.0299947 (PMC10959356; doi:10.1371/journal.pone.0299947)
Supplement: S3 Appendix — (DOCX) [file pone.0299947.s006.docx]

**S3 Appendix. List of Abbreviations**

**IRT**: Item Response Theory;

**ML**: Machine Learning;

**KNN**: K-Nearest Neighborhood;

**CV**: Cross-Validation;

**AUC**: Area Under the receiver operating characteristic Curve;

**SMOTE**: Synthetic Minority Over-Sampling Technique;

**CSROs**: Children Self-Reported Outcomes;

**PPROs**: Parent Proxy-Reported Outcomes;

**AC**: Active Caries;

**RFUTN**: Referral for Urgent Treatment Needs;

**CFA**: Confirmatory Factor Analysis;

**CFI**: Comparative Fit Index;

**TLI**: Tucker-Lewis Index;

**RMSEA**: Root Mean Square Error of Approximation;

**WLSMV**: Mean- and Variance- Adjusted Weighted Least Square;

**GRM**: Graded Response Model;

**DIF**: Differential Item Functioning;

**SVM**: Support Vector Machine;

**ROC**: Receiver Operating Characteristic Curve;

**nCV**: Nested Cross-Validation;

**AC-CSRO:** children self-reported active caries;

**RFUTN-CSRO**: children self-reported urgent treatment needs;

**AC-PPRO:** parents’ perception of their children having active caries;

**RFUTN-PPRO:** parents’ perception of their children needing a referral for urgent treatment;

**AC-DEMO-CSRO**: AC-CSRO short-form items with children-reported demographic information;

**AC-DEMO-PPRO**: AC-PPRO short-form with parent-reported demographic information;

**AC-DEMO-CSRO-PPRO**: AC-CSRO and AC-PPRO short-form items with all available demographic information;

**RFUTN-DMO-CSRO**: AC-PPRO short-form items with parent-reported demographic information;

**RFUTN-DEMO-PPRO**: RFUTN-PPRO short-form items with parent-reported demographic information;

**RFUTN-DEMO-CSRO-PPRO**: RFUTN-CSRO and RFUTN-PPRO short-form items with all available demographic information;
